# Novel Real-Time Facial Wound Recovery Synthesis Using Subsurface Scattering

**DOI:** 10.1155/2014/965036

**Published:** 2014-08-12

**Authors:** Taeyoung Choi, Seongah Chin

**Affiliations:** Sungkyul University, Anyang 430-742, Republic of Korea

## Abstract

We propose a wound recovery synthesis model that illustrates the appearance of a wound healing on a 3-dimensional (3D) face. The H3 model is used to determine the size of the recovering wound. Furthermore, we present our subsurface scattering model that is designed to take the multilayered skin structure of the wound into consideration to represent its color transformation. We also propose a novel real-time rendering method based on the results of an analysis of the characteristics of translucent materials. Finally, we validate the proposed methods with 3D wound-simulation experiments using shading models.

## 1. Introduction

A variety of face models representing the optical and biological characteristics of skin have been recently developed [1–4].

Multilayer structural wound synthesis, which makes use of a facial-tissue depth map, was proposed [5]. With this model, using a hue value from the wound image, it is possible to classify a skin-tissue layer near the wound to determine its depth. The wound can then be replicated on a 3-dimensional (3D) face. However, this method only provides a depiction of the initial state of the wound. The principal idea of the H3 model, proposed by Tabatabai et al., is that the size ratio of a wound region is determined by data measured over a period of time [6]. This study made use of a theoretical approach for medical purposes only and did not include wound recovery synthesis.

The bidirectional subsurface scattering reflection distribution function (BSSRDF) methods that have been proposed required a lengthy computation time [7, 8]. Certain enhanced image space methods that operate in real time have also been presented; however, these required precomputation and provided low frames per second (fps) [9, 10]. These approaches are discussed in more detail in Section 2.

The contributions of this paper are as follows. To the best of our knowledge, there is no existing research into wound recovery synthesis that considers the shape and color variation of a wound. Therefore, in this study, we develop a graphic synthesis model that can simulate the complete wound healing process, from start to end, in real time. To achieve this, we measure the shape and depth of a healed wound from an image. We also define a new rendering method that implements a subsurface scattering effect that is able to represent translucent materials without precomputation. The subsurface color is defined according to basic principles of optical theory and is used to render faces and wounds. We use a frame rate of more than 60 fps. This is superior to most existing methods for real-time rendering.

The preliminary conference version of this paper was presented in [11]. In this study, we extend our previous research by defining a new method for rendering faces and wounds that enhances the frame rate for real-time rendering. We use this to render faces and wounds and analyze the frame rate by demonstrating that our results maintain a similar quality as other existing subsurface methods with the higher frame rate.

The remainder of the paper is organized as follows. In Section 2, related work is discussed. The implementation of the wound contraction model is described in Section 3 and the subsurface color model is explained in Section 4. In Section 5, the real-time subsurface scattering rendering function is defined. Finally, in Section 6, experimental results are presented to evaluate the validity of the proposed method.

## 2. Related Work

Snowden [12] analyzed the relative contribution of wound contraction and epithelialization to wound healing. He discovered that a wound recovers vertically, beginning at the edge. Based on this finding, another method was proposed for determining a wound shape by calculating the average amount of healing for each border [13, 14]. The size ratio of a wound region was determined by the data measured over a time period using the H3 model [6].

The bidirectional reflection distribution function (BRDF) calculates reflectivity according to the direction of the incident light on the surface and the direction of the reflected light [15]. Conversely, BSSRDF calculates the amount of reflected light near the surrounding area due to the scattering of light at the incident point. Thus, the color of objects can be recognized when there is no incident light directly on them [7]. This model is appropriate for translucent materials such as skin. A disadvantage of the BSSRDF model, however, is its high computational cost, making real-time rendering difficult to employ. Moreover, the BSSRDF model reduces the rendering time, which exaggerates the textures of translucent materials [10]. Photorealistic subsurface scattering rendering can be performed with Monte-Carlo path tracing [16]. Although these methods are physically accurate, they typically require hours to complete. Another study employed hierarchical data structures and precomputation for rendering translucent materials [9]. However, this method also incurs a high computational cost. An image space method without precomputation for rendering translucent materials can also be employed [10]. In this method, images are displayed at 30–35 fps, which is an acceptable rate for movies.

Rendering speed plays a crucial role in real-time gaming. For interactive games where response time is critical for obtaining high scores, a rate of 35 fps may not be sufficient. The influence of frame rates in shooting games was discussed in [17]. This study compared games using a rate of 30 fps to those with 60 fps and found that players obtained higher scores when playing at 60 fps. In summary, rendering speed must be enhanced to at least 60 fps to fully capture a player's interest.

## 3. Wound Contraction Model

It has been demonstrated that a wound contraction moves towards the center of the wound [12]. Further, the amount by which a wound is reduced over a given period can be determined using the hyperbolastic model H3 [6]. The complicated healing process of a flesh wound can be divided into the hemostasis, inflammatory, proliferative, and remodeling phases. Skin wounds are healed by three mechanisms, namely, extracellular matrix formation, wound contraction, and epithelialization.

**Figure 1:**
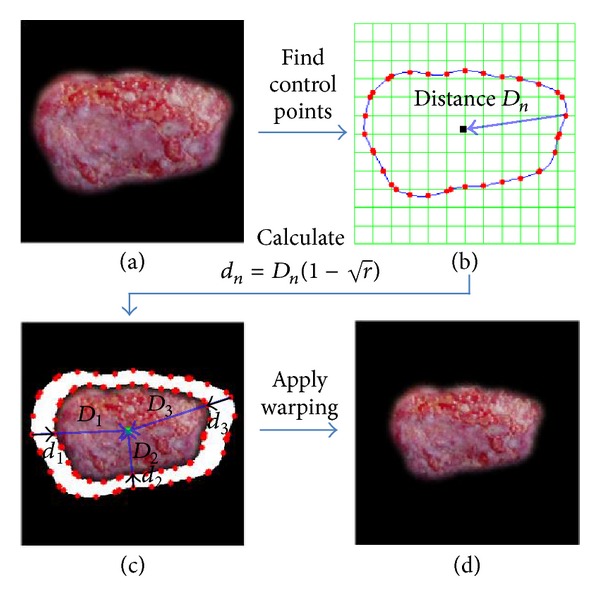
Control point sampling and wound warping process.

To simulate the contraction of a wound towards the center, we first determined the center of gravity of an input wound image. To achieve this, we used the method of the first moments [18] using each pixel value in the image. In our study, the image was binarized to calculate the center of the wound region. Then, a warping algorithm [19] was implemented to increase the contraction around the border of the wound. The control points were determined as follows. The initial wound image, illustrated in Figure 1(a), was converted into a binary format to extract the border. The border was inspected in a certain space in terms of each of the *x*- and *y*-axes, as indicated in Figure 1(b). The crossing points between the lines and the border were then established as control points.

As illustrated in Figure 1(b), *D*_*n*_ represents the distance from a control point to the first moments. In Figure 1(c), when the warping algorithm was applied by moving the extracted *n* control points in the direction of the first moments, the traveling distance *d*_*n*_ of the control points required calculation.

We consider *D*_*n*_ as a weight that is used to compute the traveling distance *d*_*n*_ of the control points from

(1) $$\begin{array}{c} d_{n}=D_{n}\left(1-\sqrt{r}\right), \end{array}$$

where *r* is the ratio of the wound size. *r* denotes the percentage of the wound that remains unhealed as determined by the H3 model.

Hence, there was no significant difference from the initial wound shape as demonstrated in Figure 1(d).

To represent the contracted wound in 3D, the depth that was calculated according to the wound location, using the face depth map of a previously developed face wound creation model [5], was employed as the initial depth of the wound. A wound recovers to normal skin by proliferation of the cells surrounding the wound [13, 14]. In this study, the H3 model [6], which calculates the extent to which a wound heals over a given period, was used for estimating the initial size of the wound.

## 4. Subsurface Color Model 

As a wound heals, its color undergoes transformation owing to changes in its composition. The color of a material can be determined by the quantity of reflective light remaining after traveling to the material, considering the amount of absorbed and scattered light, which are contingent on the depth of the material.

Human skin consists of several layers, including the epidermis, dermis, and subcutis. In this study, we used parameters such as a phase function to measure the radiance of light caused by scattering directions [20] and reflectance and transmittance ratios of the light, which are dependent on depth [21, 22].

**Figure 2:**
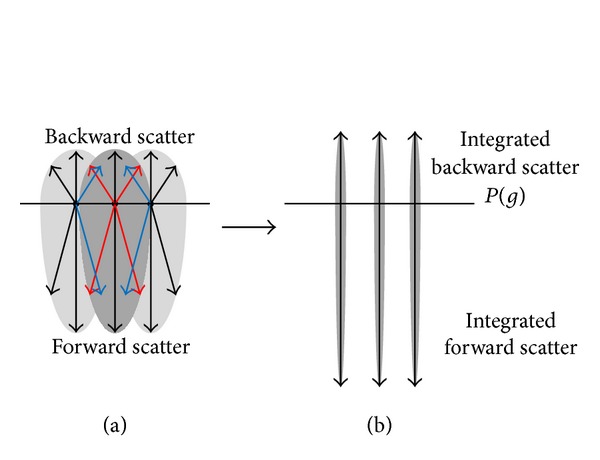
Integration of the phase function.

The change in radiance in a particular direction is caused by a loss from out-scattered light and gain from in-scattered light. As illustrated in Figure 2(a), the summation of out-scattering (red lines) and in-scattering (blue lines) involves a compromise. Hence, to design the proposed phase function, we assumed that light traversals could be classified into two groups of movement: forward scatter and backward scatter. We integrated the phase function for backward and forward scatter, the results of which are illustrated in Figure 2(b).

The refractive index of the light can be obtained using a Fresnel equation that calculates the amount of light that is refracted into the material. We employed the diffuse Fresnel reflectance *F*_*dr*_ [22] in our study.

Furthermore, we approximated the amount of light lost by computing back scatter and absorption, considering only the light inside the material. The amount of light transmission is defined by (2), which considers the depth parameter *d* as well. It can intuitively be determined that the amount of light decreases as the depth *d* increases:

(2) $$\begin{array}{c} T\left(d\right)=\left(1-\sigma_{a}d-\sigma_{s}^{\prime }P\left(g\right)d\right)\left(1-F_{dr,b}\right), \end{array}$$

where *σ*_*a*_ and *σ*_*s*_′ are the absorption and reduced scattering coefficients of the wound tissue, respectively. *P*(*g*) is the integration of the phase function for backward scatter and *d* is the depth. *F*_*dr*,*b*_ represents the diffuse Fresnel reflectance at the lower boundary. According to (2), the range of the depth *d* becomes 0 < *d* < 1/(*σ*_*a*_ + *σ*_*s*_′*P*(*g*)) because 0 ≤ *T*(*d*) ≤ 1.

As the light moves to a greater depth, the amount of light decreases as loss and absorption increase. For example, back scattered light decreases because of absorption when the light moves forward and backward inside the material. The total reflectance *R*_(*d*)_ is defined as the amount of light that moves into the subsurface and back to the surface. That is,

(3) R(d)=(1−Fdr,t)(∫0d(1−σax)2σs′P(g)dx      +Fdr,b(1−σad−σs′P(g)d)2)=(1−Fdr,t)(σs′P(g){d−σad2+13σa2d3}      +Fdr,b(1−σad−σs′P(g)d)2),

where *F*_*dr*,*t*_ represents the diffuse Fresnel reflectance at the upper boundary.

**Figure 3:**
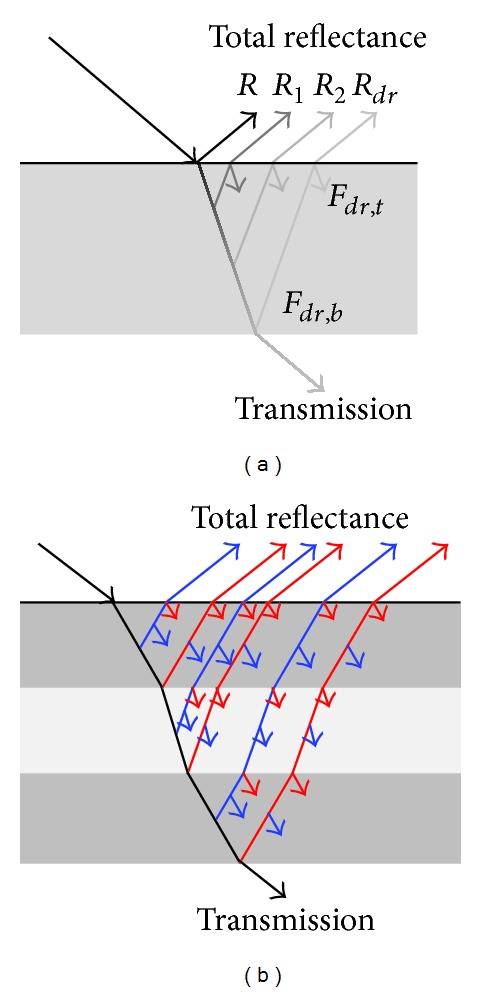
Reflectance and transmission model for (a) single layer and (b) multilayers.

To facilitate understanding transmission and total reflectance, we display the proposed approach in a single-layered material in Figure 3(a). *R*_1_ and *R*_2_ represent the reflectance due to subsurface scattering and absorption. Whereas we conceptually visualize subsurface scattering in a discrete manner, technically, scattering levels are computed continuously using mathematical integration.

*R*_*dr*_ represents the reflectance caused by the bottom reflection. Given a light vector, the reflectance *R* and the transmission on the surface can be calculated using Ward's BRDF model [15] or other reflection models. The back scattering (blue line) and reflection (red line) components of each layer are indicated in Figure 3(b). The single-layered model can be extended to the multilayered model by considering the amount of transmission at the first layer to be the quantity of incoming light at the second layer. To obtain the total reflectance at the first and second layers, we add *F*_*dr*,*b*_ at the first layer to the total reflectance at the second layer.

To represent the total reflectance of the wound recovery, the depths of the three layers of the skin, namely, the epidermis, dermis, and subcutis, must be considered. The depths of the wound in the middle of the recovery process follow the same velocity as described in Section 3.

In this study, we have modified the skin parameters that were originally introduced by Tuchin [23]. The values of the parameters are significantly optimized by the use of the facial depth map [5] presented in Table 1.

To achieve a high-quality wound synthesis representation, we implement a blending function that employs wound texture, proposed reflectance, and skin texture when rendering the final appearance of a recovered wound. The deviation *D* between the total reflectance *R*_(*d*)_ conveying the colors when the recovery is complete and the skin texture is optimized by the recovery ratio *r*. The subsurface color of the recovering wound is defined according to the following blending function:

(4) $$\begin{array}{c} C=\left(1-0.8\sqrt{r}\right)W+0.8\sqrt{r}R_{\left(d\right)}+0.4rD, \end{array}$$

where *C* is the subsurface color of the recovering wound, *W* is the texture map of the wound, *r* is the recovery ratio, *R*_(*d*)_ is the multilayered total reflectance computed using (3), and *D* is the deviation between the skin texture and the total reflectance.

## 5. Real-Time Rendering

The colors of objects that can be recognized by the human eye consist of the sum of the emissive, ambient, diffuse, and specular colors. Of these, the emissive and ambient colors are those that are visible in a given object. The diffuse and specular colors are reflected in areas where light is directly incident. However, in real objects, materials that have translucent characteristics can reflect light even in areas that do not receive direct light [15, 22]. To represent this phenomenon, the real-time subsurface scattering rendering function defined and used in this paper makes use of three concepts: surface diffuse, subsurface scattering diffuse, and specular.

Our model uses the Lambert diffuse light as the surface diffuse. A novel method is used for the subsurface scattering diffuse to represent the translucent effect. Finally, the specular uses the specular light of Ward's BRDF.

The Lambert diffuse light can be determined easily by the inner product of the incident vector of the light *L* and the normal vector of a surface *N*, which is expressed as in the following:

(5) $$\begin{array}{c} {\text{Diffuse}}_{\text{surface}}\left(L,N\right)=L\cdot N. \end{array}$$

The subsurface scattering diffuse light model is a rendering model that is used to represent the translucent nature of materials. This model employs the characteristics that are generally exhibited by a rendering model when a light source is directed at an object. If the light is positioned further away from the object, the brightness is inversely proportional to the distance from the light source to the position of the object. For instance, for a representative dipole model, BSSRDF showed that the distribution of radiance becomes inversely proportional to the distance from the light [13]. In place of using the distance from an incident point to an outgoing point on a surface, we used the distance from the light source to the incident point and from the light source to the outgoing point.

Therefore, the subsurface scattering diffuse light model, used to represent translucent materials, was determined using the distance between the location of the light source and the location of each object. The rendering results of BSSRDF showed a reduction in light when the location of the light source was further away from the location of each area [10]. The subsurface scattering diffuse light model is given by

(6) $$\begin{array}{c} {\text{Diffuse}}_{\text{subsurface}}\left(d\right)=\frac{1}{{\left(\alpha d+\beta \right)}^{\gamma }}, \end{array}$$

where *d* is the distance to a light source and *α*,  *β*, and *γ* are subsurface diffuse light coefficients. These variables differ according to the size and center of an object.

The specular value of Ward's BRDF, which is an anisotropic reflection model that uses Gaussian reflectance instead of the Phong model, is used as the specular. This model uses two angles, namely, the azimuth angle and the elevation angle, for the incident and reflection, respectively, as parameters [15]. Equation (7) shows only the specular section of Ward's BRDF:

(7) Specular(θi,ϕi;θr,ϕr) =Icos⁡θ(ρs4παxαycos⁡θicos⁡θre−tan2δ(cos⁡2ϕ/αx2+sin2ϕ/αy2)),

where (*θ*_*i*_, *ϕ*_*i*_) is the incident light vector and (*θ*_*r*_, *ϕ*_*r*_) is the reflected light vector. *I* represents the light radiance and *θ* is the angle between the normal vector and the light vector. *ρ*_*s*_ represents the specular coefficient, *α*_*x*_ and *α*_*y*_ are the standard deviations of the surface slope in the *x* and *y* directions, and *δ* is the angle between the normal vector *N* and the half vector *H*. Furthermore, *ϕ* is the azimuth angle of the half vector that is projected onto the surface. The final rendering color *C*_*L*_, which combines (5), (6), and (7) and the subsurface color of a recovery wound *C*, is defined as

(8) CL=C(ρdcos⁡θ+ρts(αd+β)γ)+Icos⁡θ ×(ρs4παxαycos⁡θicos⁡θre−tan2δ(cos⁡2ϕ/αx2+sin2ϕ/αy2)),

where *C* is the subsurface color from (4) and *ρ*_*d*_, *ρ*_ts_, and *ρ*_*s*_ are the diffuse, translucent, and specular coefficients, respectively. The coefficients can be optimized with experimentation.

## 6. Experimental Results

Facial wound contraction and the models of color transformation over a given period were implemented using Microsoft's MFC, the OpenGL API, and a CG shader with an Intel Core i3 CPU 550 at 3.20 GHz and NVDIA GeForce GTX 580. Our 3D model is composed of 7,344 meshes with 14,432 vertices for the head and 6,336 meshes with 3,330 vertices for the frontal face.

### 6.1. Validation of Wound Contraction

**Figure 4:**
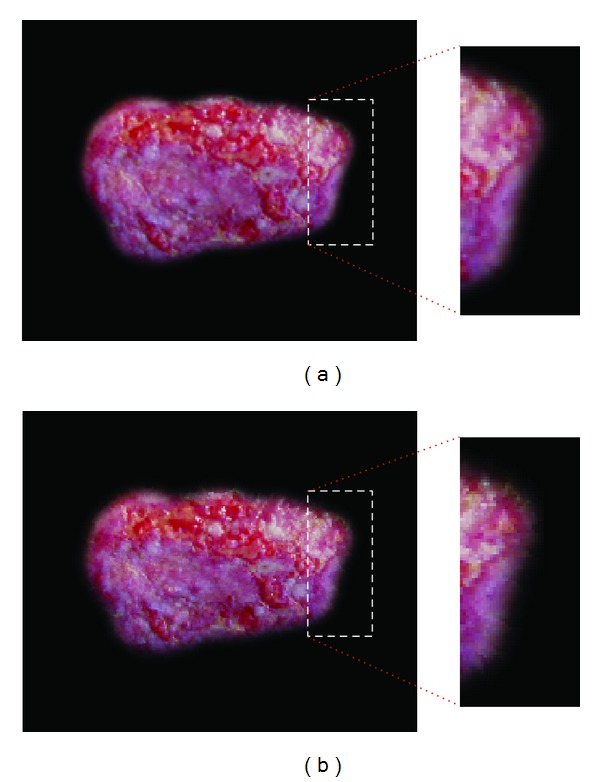
Shapes of contracted wounds using (a) the image scaling method and (b) the weighted warping method.

**Figure 5:**
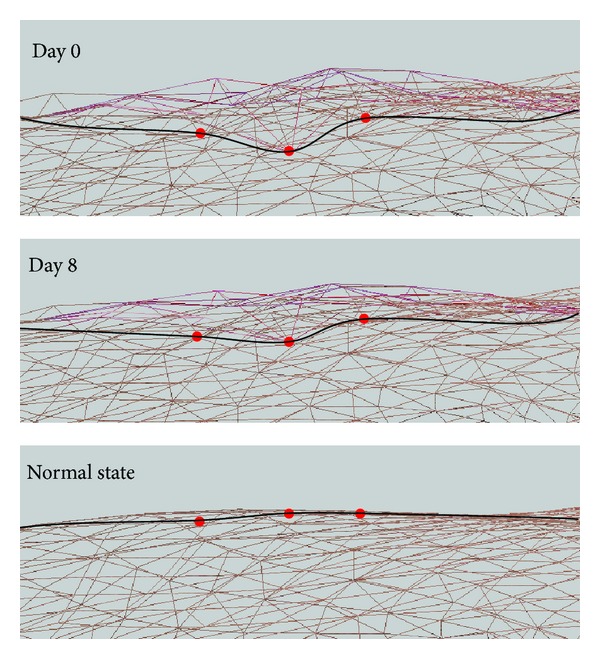
Change in depth as the wound healed.

In our experiment, the amount of wound recovery that occurred in a given period was determined using the H3 model, and the contraction and depth transformation were performed using the methods proposed in this paper. The experimental results are illustrated in Figures 4 and 5.

In Figure 4, the 60% contraction results for an initial wound image, using (a) the image scaling method and (b) the weighted warping method, are presented. It can be observed from Figure 4(a) that the size of the wound was smaller than that of the initial wound. However, the borders and the inside were found to be the same as those of the initial wound. In contrast, Figure 4(b) illustrates that whereas the shape of the borders did not change significantly because of the application of weights according to the distance between a control point and the center point, the borders of the wound exhibited visible tension where control points caused increased contraction related to the warping algorithm.

In each image of Figure 5, the red dots and black lines represent the depth of the wound. It can be seen that the depth of the wound was gradually reduced from day 0 to become normal skin.

### 6.2. Validation of Wound Color and Real-Time Subsurface Scattering Rendering

**Figure 6:**
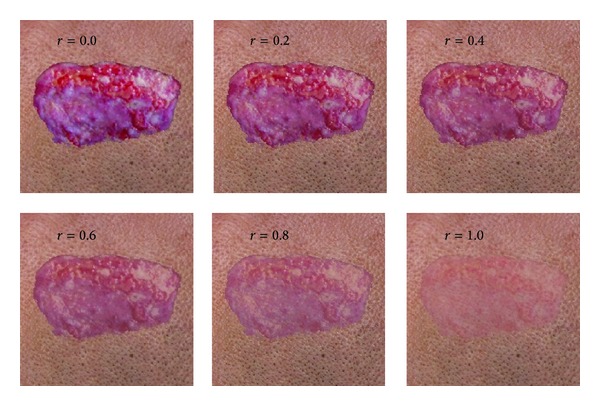
Color transformation of a wound with increasing recovery rate.

To validate the proposed subsurface color model, we first computed the reflectance of each layer using (3). To illustrate the wound recovery more effectively, we used the blending function given by (4). The color transformation during the wound recovery that represents only colors and not shapes is presented in Figure 6. We discovered that the wound color converged to the skin color as the wound healed.

**Figure 7:**
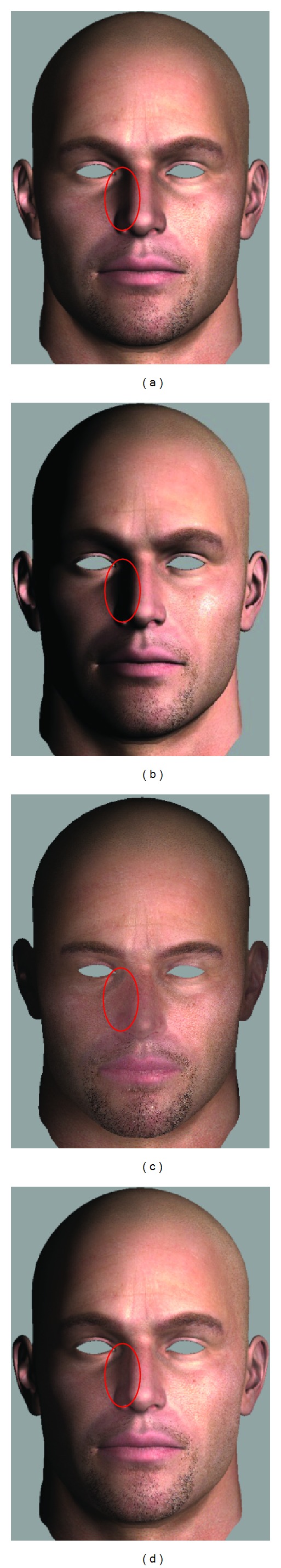
Rendering results of (a) Ward's BRDF, (b) the layered Oren-Nayar BRDF, (c) Chang's texture space BSSRDF, and (d) our real-time subsurface scattering rendering.

The most significant difference between the proposed real-time rendering model and the existing rendering models is the translucent effect.  Figure 7 presents the results of (a) Ward's BRDF [15], (b) the layered Oren-Nayar method [24], (c) Chang's texture space BSSRDF [25], and (d) the proposed real-time subsurface scattering. The light source was located on the right in all representations. Therefore, because the right-hand side of the nose was on the opposite side of the light source, as indicated by the red circles, it received no direct incident light and was displayed as a black color in these models. However, in Chang's BSSRDF (c) and the proposed real-time rendering (d), the translucent effect was illustrated, because the light was diffused around the adjacent area.

### 6.3. Rendering Results

**Figure 8:**
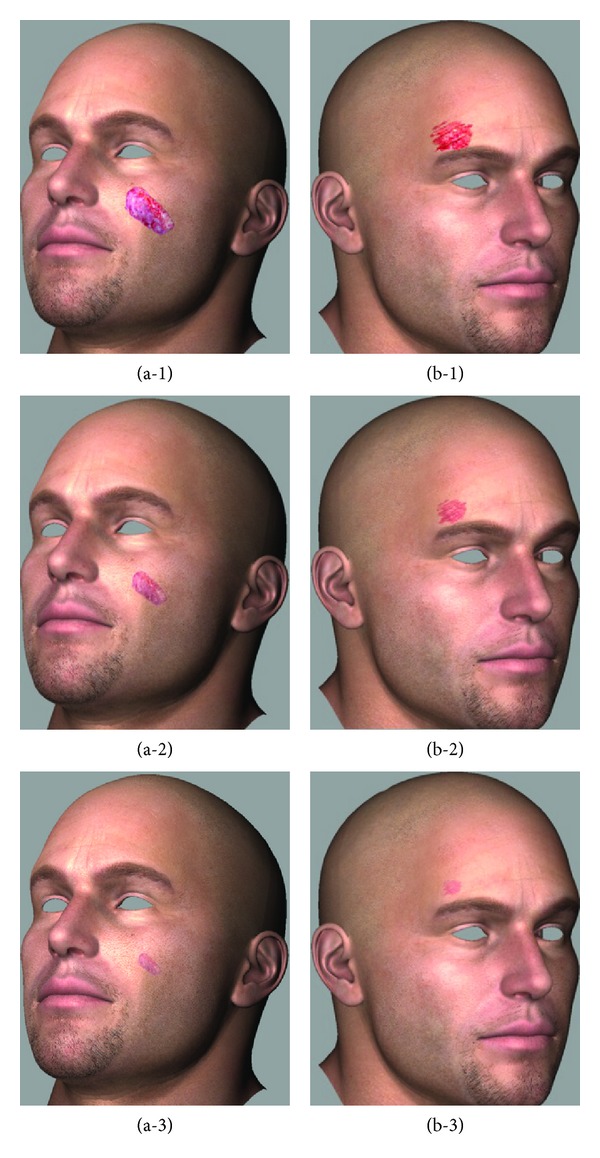
Rendering results showing the process of wound recovery synthesis.

**Figure 9:**
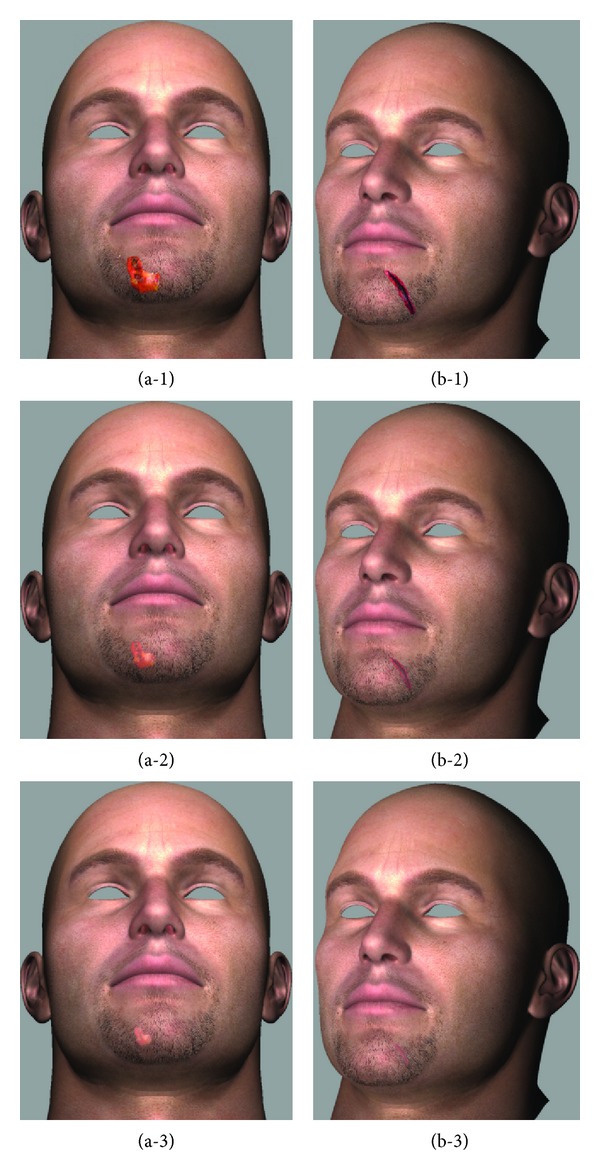
Results of rendering of the chin.

A predeveloped wound synthesis using a 3D face was used to illustrate the color transformation of a contracted wound based on a wound image [5]. Furthermore, we demonstrated the proposed real-time subsurface scattering rendering of wound synthesis based on the position of the light source. In the rendering experiment, the distance between the center of the object and the light source was 2 and the size of the object was 2.2. The parameters used in the experiment were as follows: *α* = 0.2,  *β* = 0.75,  *γ* = 10 and *ρ*_*d*_ = 0.7,  *ρ*_*s*_ = 0.8,  *ρ*_ts_ = 0.7,  *α*_*x*_ = *α*_*y*_ = 0.4. The application of the shading model is shown in Figures 8 and 9.

The initial wounds on the cheek and above the eyebrow are illustrated in Figures 8 (a-1) and (b-1), and the wound contractions using the method described in Sections 3 and 4 after day 10 are presented in Figures 8 (a-2) and (b-2). Figures 8 (a-3) and (b-3) show the wounds after day 12. As can be seen in the figures, the effect of the light source in the shading model was verified by the wound images of each phase.

Moreover, the effectiveness of representing a wound that has a different shape and color was demonstrated using the various wound images.  Figure 9 shows the results of the shape and color transformation according to the healing of the wounds on the chin in (a) and (b).

### 6.4. Validation of Frame Rate

To validate the proposed frame rate, we surveyed the frame rates of other BRDF and BSSRDF methods. BSSRDF methods did not satisfy the minimal frame rate of 60 fps required by real-time games to ensure effective player interaction [17]. Wang's BSSRDF frame rate had a maximum of 31.8 fps [9]. Chang's BSSRDF frame rate did not surpass 20 fps. The methods of Li and Shat et al. achieved only 10 fps and 31 fps, respectively. Conversely, Ward's [15] and the layered Oren-Nayar [24] BRDF methods showed better frame rates (115-116 fps) than the BSSRDF methods. Similar to the BRDF methods, the proposed method achieved 114 fps and was capable of representing subsurface scattering effects.

## 7. Conclusion

In this paper, we proposed models for representing color and shape transformations during wound recovery and real-time subsurface scattering. The change ratio of the wound recovery was derived from the H3 model. A warping algorithm was used to represent the contraction of a wound. Further, we presented our color reflectance model that considers the subsurface scattering of the wound due to the multilayered skin structure. We successfully performed rendering of the translucent effect in real time. A wound will leave a scar based on the initial wound shape and in general will gradually return to normal skin throughout the remodeling phase. Therefore, it is necessary for a model for scars that remain after wounds have healed to be developed in the future.

## Figures and Tables

**Table 1:** Optical parameters for facial skin.

	Epidermis	Dermis	Subcutis
*σ*_*a*_			
R	0.04	0.001	0.078
G	1.44	0.232	0.75
B	2.08	0.239	0.53
*σ*_*s*_′			
R	10.5	7	5.9
G	11	7.3	6.3
B	11.6	7.5	6.7
*η*	1.5	1.4	1.4
*g*	0.2	0.76	0.73
*d* (mm)	0.2	1~4	4~9
